# Diaqua­bis[2-(benzyl­oxy)acetato]cobalt(II)

**DOI:** 10.1107/S1600536808016899

**Published:** 2008-06-07

**Authors:** Chun-Liang Chen, Sheng-Li Sun, Chang-Sheng Gu, Weng-Dong Song, Xiao-Min Hao

**Affiliations:** a Monitoring Center of Marine Resources and the Environment, Guangdong Ocean University, Zhanjiang 524088, People’s Republic of China; b Department of Applied Chemistry, Guangdong Ocean University, Zhanjiang 524088, People’s Republic of China

## Abstract

In the mononuclear title complex, [Co(C_9_H_9_O_3_)_2_(H_2_O)_2_], each Co^II^ atom is located on an inversion center and is hexa­coordinated by four O atoms from two benzyl­oxyacetate ligands [Co—O bond lengths = 2.0487 (9) and 2.1090 (9) Å] and two water mol­ecules [Co—O bond length = 2.0873 (9) Å] in a distorted octa­hedral geometry. In the crystal structure, inter­molecular hydrogen bonds and π–π stacking inter­actions [centroid–centroid distance between phenyl rings = 3.692 (2) Å] link the mol­ecules into a supra­molecular structure.

## Related literature

For the crystal structure of a similar Cu^II^ complex of benzyl­oxyacetate, see: Sun *et al.* (2008 ▶).
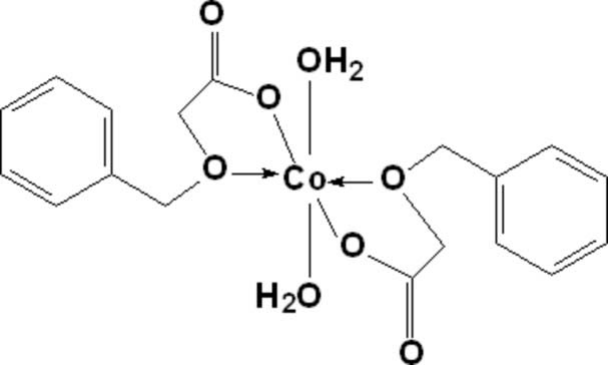


## Experimental

### 

#### Crystal data

[Co(C_9_H_9_O_3_)_2_(H_2_O)_2_]
*M* *_r_* = 425.29
Monoclinic, 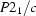

*a* = 11.4968 (1) Å
*b* = 7.1557 (1) Å
*c* = 12.0054 (1) Å
β = 109.708 (1)°
*V* = 929.80 (2) Å^3^
*Z* = 2
Mo *K*α radiation
μ = 0.97 mm^−1^
*T* = 296 (2) K
0.32 × 0.26 × 0.18 mm

#### Data collection

Bruker P4 diffractometer
Absorption correction: multi-scan (*SADABS*; Sheldrick, 2000 ▶) *T*_min_ = 0.743, *T*_max_ = 0.835
7958 measured reflections
2126 independent reflections
1904 reflections with *I* > 2σ(*I*)
*R*_int_ = 0.020

#### Refinement

*R*[*F* ^2^ > 2σ(*F* ^2^)] = 0.023
*wR*(*F* ^2^) = 0.066
*S* = 1.07
2115 reflections
131 parameters
3 restraints
H atoms treated by a mixture of independent and constrained refinement
Δρ_max_ = 0.27 e Å^−3^
Δρ_min_ = −0.23 e Å^−3^

### 

Data collection: *APEX2* (Bruker, 2004 ▶); cell refinement: *SAINT* (Bruker, 2004 ▶); data reduction: *SAINT*; program(s) used to solve structure: *SHELXS97* (Sheldrick, 2008 ▶); program(s) used to refine structure: *SHELXL97* (Sheldrick, 2008 ▶); molecular graphics: *SHELXTL* (Sheldrick, 2008 ▶); software used to prepare material for publication: *SHELXL97*.

## Supplementary Material

Crystal structure: contains datablocks I, global. DOI: 10.1107/S1600536808016899/lx2056sup1.cif

Structure factors: contains datablocks I. DOI: 10.1107/S1600536808016899/lx2056Isup2.hkl

Additional supplementary materials:  crystallographic information; 3D view; checkCIF report

## Figures and Tables

**Table 1:** Hydrogen-bond geometry (Å, °)

*D*—H⋯*A*	*D*—H	H⋯*A*	*D*⋯*A*	*D*—H⋯*A*
O1*W*—H1*W*1⋯O2^i^	0.84 (2)	1.94 (1)	2.768 (2)	169 (2)
O1*W*—H1*W*2⋯O2^ii^	0.846 (9)	1.94 (1)	2.773 (1)	171 (2)

Symmetry codes: (i) 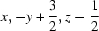; (ii) 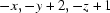.

**Fig. 1.**
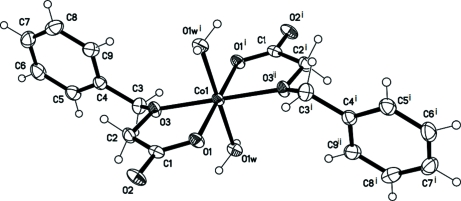
Molecular structure of (I) with 30% probability ellipsoids. [Symmetry code: (i) -x, -y+1, -z+1.]

**Fig. 2.**
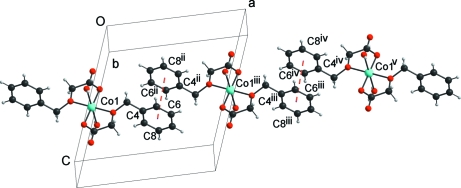
π—π interaction (dotted lines) in the title compound. Cg denotes the ring centroid. [Symmetry code: (ii) -x+1, -y+1, -z+1; (iii) x+1, y , z; (iv) -x+2, -y+1, -z+1; (v) x+2, y, z.]

## supplementary crystallographic information

### Comment

Recently, we have reported the crystal structure of the complex of benzyloxyacetate, [Cu(C_9_H_9_O_3_)_2_.2(H_2_O)], (Sun *et al.*, 2008). Here we report the crystal structure of the title mononuclear complex of benzyloxyacetate, [Co(C_9_H_9_O_3_)_2_.2(H_2_O)], (Fig. 1).

The structure of the title compound is similar to that of the Cu(II) complex (Sun *et al.*,  2008). The Co atom lies on an inversion center and displays an octahedral geometry defined by two carboxylate O atoms and two benzyloxy O atoms from two benzyloxyacetate ligands, and two water molecules, respectively. The Co—O and Co—Ow bond lengths are 2.0487 (9), 2.1090 (9) and 2.0873 (9) Å, respectively. The characteristic C—O(carboxylate) bond lengths suggest electron localization of the carboxylate groups of the anionic ligands. The molecular packing is stabilized by intermolecular O—H···O hydrogen bond interactions (Table 1). The crystal packing (Fig. 2) is further stabilized by aromatic π—π stacking interaction between the benzene ring from neighbouring molecules. The Cg···Cg^ii^ distance is 3.692 (2) Å (*Cg* is the centroids of the C4-C9 benzene ring, symmetry code as in Fig. 2).

### Experimental

The ligand, benzyloxyacetic acid was commercially available and used without further purification. The title complex was prepared by the addition of Cobalt diacetate trihydrate (2.38 g, 10 mmol) to a hot aqueous solution of benzyloxyacetic acid (1.66 g, 10 mmol); the pH was adjusted to 6 with 0.1*M* sodium hydroxide. The solution was allowed to evaporate at room temperature. Pink prismatic crystals separated from the filtered solution after several days. C&H analysis. Calc. for C_18_H_22_CoO_8_: C 50.83, H 5.21%. Found: C 50.81, H 5.22%.

### Refinement

The H atoms were placed in calculated positions, with C—H = 0.93 or 0.97 Å, and *U*_iso_(H) = 1.2*U*_eq_(C), and were refined in the riding-model approximation. The H atoms of the water molecule were was located in a difference Fourier map and refined with O—H = 0.85Å and *U*_iso_(H) = 1.5*U*_eq_(O).

### Crystal data

**Table:**

[Co(C_9_H_9_O_3_)_2_(H_2_O)_2_]	*F*_000_ = 442
*M**_r_* = 425.29	*D*_x_ = 1.519 Mg m^−^^3^
Monoclinic, *P*2_1_/*c*	Mo *K*α radiation λ = 0.71073 Å
Hall symbol: -P 2ybc	Cell parameters from 7958 reflections
*a* = 11.4968 (1) Å	θ = 1.9–27.5º
*b* = 7.1557 (1) Å	µ = 0.97 mm^−^^1^
*c* = 12.0054 (1) Å	*T* = 296 (2) K
β = 109.708 (1)º	Prism, pink
*V* = 929.80 (2) Å^3^	0.32 × 0.26 × 0.18 mm
*Z* = 2	

### Data collection

**Table:**

Bruker P4 diffractometer	2126 independent reflections
Radiation source: fine-focus sealed tube	1904 reflections with *I* > 2σ(*I*)
Monochromator: graphite	*R*_int_ = 0.020
Detector resolution: 10.000 pixels mm^-1^	θ_max_ = 27.5º
*T* = 296(2) K	θ_min_ = 1.9º
ω scans	*h* = −14→14
Absorption correction: multi-scan(SADABS; Sheldrick, 2000)	*k* = −9→8
*T*_min_ = 0.743, *T*_max_ = 0.835	*l* = −15→15
7958 measured reflections	

### Refinement

**Table:**

Refinement on *F*^2^	Secondary atom site location: difference Fourier map
Least-squares matrix: full	Hydrogen site location: inferred from neighbouring sites
*R*[*F*^2^ > 2σ(*F*^2^)] = 0.023	H atoms treated by a mixture of independent and constrained refinement
*wR*(*F*^2^) = 0.066	*w* = 1/[σ^2^(*F*_o_^2^) + (0.0357*P*)^2^ + 0.2056*P*] where *P* = (*F*_o_^2^ + 2*F*_c_^2^)/3
*S* = 1.07	(Δ/σ)_max_ < 0.001
2115 reflections	Δρ_max_ = 0.27 e Å^−^^3^
131 parameters	Δρ_min_ = −0.23 e Å^−^^3^
3 restraints	Extinction correction: SHELXL, Fc^*^=kFc[1+0.001xFc^2^λ^3^/sin(2θ)]^-1/4^
Primary atom site location: structure-invariant direct methods	Extinction coefficient: 0.042 (3)

### Fractional atomic coordinates and isotropic or equivalent isotropic displacement parameters (Å^2^)

**Table:**

	*x*	*y*	*z*	*U*_iso_*/*U*_eq_	
Co1	0.0000	0.5000	0.5000	0.02597 (10)	
O1W	−0.07843 (9)	0.62124 (13)	0.33323 (8)	0.0367 (2)	
O1	−0.04160 (8)	0.73017 (12)	0.58087 (8)	0.0344 (2)	
O2	0.02557 (13)	1.00099 (11)	0.66922 (10)	0.0431 (3)	
O3	0.15775 (8)	0.66897 (13)	0.52873 (8)	0.0349 (2)	
C1	0.03731 (12)	0.85740 (17)	0.61469 (10)	0.0309 (3)	
C2	0.15726 (12)	0.83967 (19)	0.58941 (12)	0.0363 (3)	
C3	0.24243 (12)	0.6638 (2)	0.46413 (12)	0.0395 (3)	
C4	0.37510 (12)	0.66780 (18)	0.54400 (12)	0.0339 (3)	
C5	0.46331 (15)	0.7544 (2)	0.50689 (15)	0.0433 (3)	
C6	0.58642 (15)	0.7538 (2)	0.57881 (19)	0.0553 (4)	
C7	0.62120 (15)	0.6693 (2)	0.68845 (17)	0.0549 (4)	
C8	0.53398 (15)	0.5825 (3)	0.72509 (15)	0.0523 (4)	
C9	0.41170 (14)	0.5813 (2)	0.65364 (13)	0.0433 (3)	
H2A	0.1659	0.9441	0.5413	0.044*	
H2B	0.2264	0.8420	0.6631	0.044*	
H3A	0.2269	0.7701	0.4110	0.047*	
H3B	0.2279	0.5512	0.4165	0.047*	
H5	0.4400	0.8132	0.4335	0.052*	
H6	0.6456	0.8105	0.5531	0.066*	
H7	0.7035	0.6711	0.7373	0.066*	
H8	0.5575	0.5241	0.7986	0.063*	
H9	0.3533	0.5219	0.6792	0.052*	
H1W1	−0.0549 (19)	0.573 (2)	0.2805 (15)	0.065*	
H1W2	−0.0696 (19)	0.7387 (13)	0.3341 (17)	0.065*	

### Atomic displacement parameters (Å^2^)

**Table:**

	*U*^11^	*U*^22^	*U*^33^	*U*^12^	*U*^13^	*U*^23^
Co1	0.02885 (15)	0.01952 (14)	0.03148 (15)	−0.00140 (8)	0.01273 (10)	−0.00332 (8)
O1W	0.0462 (5)	0.0281 (5)	0.0367 (5)	0.0011 (4)	0.0153 (4)	0.0015 (4)
O1	0.0407 (5)	0.0245 (4)	0.0436 (5)	−0.0009 (4)	0.0216 (4)	−0.0046 (4)
O2	0.0696 (7)	0.0230 (5)	0.0448 (6)	−0.0019 (4)	0.0299 (5)	−0.0070 (4)
O3	0.0326 (4)	0.0299 (5)	0.0470 (5)	−0.0057 (4)	0.0197 (4)	−0.0106 (4)
C1	0.0446 (7)	0.0220 (6)	0.0275 (5)	0.0022 (5)	0.0139 (5)	0.0011 (5)
C2	0.0383 (7)	0.0265 (6)	0.0434 (7)	−0.0058 (5)	0.0126 (6)	−0.0080 (5)
C3	0.0362 (7)	0.0477 (8)	0.0386 (7)	−0.0027 (6)	0.0178 (5)	−0.0039 (6)
C4	0.0347 (6)	0.0295 (6)	0.0409 (7)	−0.0001 (5)	0.0170 (5)	−0.0025 (5)
C5	0.0432 (7)	0.0376 (8)	0.0559 (9)	−0.0003 (6)	0.0258 (7)	0.0035 (6)
C6	0.0400 (8)	0.0425 (9)	0.0919 (13)	−0.0059 (7)	0.0333 (8)	−0.0052 (9)
C7	0.0360 (7)	0.0473 (9)	0.0726 (11)	0.0065 (7)	0.0068 (7)	−0.0131 (8)
C8	0.0505 (9)	0.0526 (10)	0.0488 (8)	0.0164 (8)	0.0101 (7)	0.0011 (7)
C9	0.0440 (8)	0.0403 (8)	0.0491 (8)	0.0040 (6)	0.0202 (6)	0.0056 (7)

### Geometric parameters (Å, °)

**Table:**

Co1—O1	2.0487 (9)	C3—C4	1.503 (2)
Co1—O3	2.1090 (9)	C3—H3A	0.9700
Co1—O1W	2.0873 (9)	C3—H3B	0.9700
O1—C1	1.252 (2)	C4—C5	1.384 (2)
O2—C1	1.250 (2)	C4—C9	1.385 (2)
Co1—O1^i^	2.0487 (9)	C5—C6	1.387 (2)
Co1—O3^i^	2.1090 (9)	C5—H5	0.9300
Co1—O1W^i^	2.0873 (9)	C6—C7	1.380 (3)
O3—C2	1.423 (2)	C6—H6	0.9300
O3—C3	1.435 (2)	C7—C8	1.372 (3)
O1W—H1W1	0.84 (2)	C7—H7	0.9300
O1W—H1W2	0.846 (9)	C8—C9	1.378 (2)
C1—C2	1.514 (2)	C8—H8	0.9300
C2—H2A	0.9700	C9—H9	0.9300
C2—H2B	0.9700		
			
O1—Co1—O1^i^	180.0	C1—C2—H2B	109.7
O1—Co1—O3	77.70 (3)	C2—O3—C3	114.77 (10)
O1—Co1—O3^i^	102.30 (3)	C2—O3—Co1	115.10 (7)
O1—Co1—O1W	91.49 (4)	C3—O3—Co1	126.84 (8)
O1—Co1—O1W^i^	88.51 (4)	C4—C3—H3A	109.1
O3^i^—Co1—O3	180.00 (7)	C4—C3—H3B	109.1
O1W—Co1—O3^i^	90.68 (4)	C4—C5—C6	120.18 (15)
O1W—Co1—O3	89.32 (4)	C4—C5—H5	119.9
O1W^i^—Co1—O1W	180.0	C4—C9—H9	119.7
Co1—O1W—H1W1	114.1 (14)	C5—C4—C9	119.03 (13)
Co1—O1W—H1W2	112.9 (13)	C5—C4—C3	119.94 (13)
O1^i^—Co1—O3	102.30 (3)	C5—C6—H6	119.9
O1^i^—Co1—O3^i^	77.70 (3)	C6—C5—H5	119.9
O1^i^—Co1—O1W^i^	91.49 (4)	C6—C7—H7	120.1
O1^i^—Co1—O1W	88.51 (4)	C7—C8—C9	120.38 (16)
O1W^i^—Co1—O3^i^	89.32 (4)	C7—C8—H8	119.8
O1W^i^—Co1—O3	90.68 (4)	C7—C6—C5	120.12 (15)
O1—C1—C2	118.90 (11)	C7—C6—H6	119.9
O2—C1—O1	124.96 (13)	C8—C7—C6	119.76 (15)
O2—C1—C2	116.14 (12)	C8—C7—H7	120.1
O3—C2—C1	109.61 (10)	C8—C9—C4	120.52 (15)
O3—C2—H2A	109.7	C8—C9—H9	119.7
O3—C2—H2B	109.7	C9—C4—C3	121.01 (12)
O3—C3—C4	112.47 (11)	C9—C8—H8	119.8
O3—C3—H3A	109.1	H1W1—O1W—H1W2	110.4 (14)
O3—C3—H3B	109.1	H2A—C2—H2B	108.2
C1—O1—Co1	118.60 (8)	H3A—C3—H3B	107.8
C1—C2—H2A	109.7		
			
O1W^i^—Co1—O1—C1	88.19 (9)	Co1—O3—C2—C1	−1.77 (13)
O1W—Co1—O1—C1	−91.81 (9)	O2—C1—C2—O3	179.19 (11)
O3^i^—Co1—O1—C1	177.16 (9)	O1—C1—C2—O3	−0.56 (17)
O3—Co1—O1—C1	−2.84 (9)	C2—O3—C3—C4	67.25 (15)
O1—Co1—O3—C2	2.39 (9)	C1—O3—C3—C4	90.21 (17)
O1^i^—C1—O3—C2	−177.45 (11)	O3—C3—C4—C5	−147.57 (13)
O1W^i^—Co1—O3—C2	−85.93 (9)	O3—C3—C4—C9	34.23 (19)
O1W—Co1—O3—C2	94.07 (9)	C9—C4—C5—C6	0.1 (2)
O1—Co1—O3—C3	−155.88 (11)	C3—C4—C5—C6	−178.14 (14)
O1^i^—Co1—O3—C3	24.12 (11)	C4—C5—C6—C7	−1.0 (2)
O1W^i^—Co1—O3—C3	115.79 (11)	C5—C6—C7—C8	1.3 (3)
O1W—Co1—O3—C3	−64.21 (11)	C6—C7—C8—C9	−0.8 (3)
Co1—O1—C1—O2	−176.97 (10)	C7—C8—C9—C4	−0.1 (3)
Co1—O1—C1—C2	2.76 (15)	C5—C4—C9—C8	0.4 (2)
C3—O3—C2—C1	159.19 (11)	C3—C4—C9—C8	178.66 (14)

Symmetry codes: (i) −*x*, −*y*+1, −*z*+1.

### Hydrogen-bond geometry (Å, °)

**Table:**

*D*—H···*A*	*D*—H	H···*A*	*D*···*A*	*D*—H···*A*
O1W—H1W1···O2^ii^	0.84 (2)	1.94 (1)	2.768 (2)	169 (2)
O1W—H1W2···O2^iii^	0.846 (9)	1.94 (1)	2.773 (1)	171 (2)

Symmetry codes: (ii) *x*, −*y*+3/2, *z*−1/2; (iii) −*x*, −*y*+2, −*z*+1.
